# Modeling the space-time correlation of pulsed twin beams

**DOI:** 10.1038/s41598-023-42588-y

**Published:** 2023-10-05

**Authors:** Alessandra Gatti, Ottavia Jedrkiewicz, Enrico Brambilla

**Affiliations:** 1 Istituto di Fotonica e Nanotecnologie del CNR, Piazza Leonardo da Vinci 32, 20133 Milano, Italy; 2 Dipartimento di Scienze e Alta Tecnologia, Università dell’Insubria, Via Valleggio 11, 22100 Como, Italy

**Keywords:** Quantum optics, Quantum simulation

## Abstract

Entangled twin-beams generated by parametric down-conversion are among the favorite sources for imaging-oriented applications, due their multimodal nature in space and time. However, a satisfactory theoretical description is still lacking. In this work we propose a semi-analytic model which aims to bridge the gap between time-consuming numerical simulations and the unrealistic plane-wave pump theory. The model is used to study the quantum correlation and the coherence in the angle-frequency domain of the parametric emission, and demonstrates a $g^{{1/2}}$ growth of their size as the gain *g* increases, with a corresponding contraction of the space-time distribution. These predictions are systematically compared with the results of stochastic numerical simulations, performed in the Wigner representation, of the full model equations: an excellent agreement is shown even for parameters well outside the expected limit of validity of the model.

## Introduction

**Figure 1:**
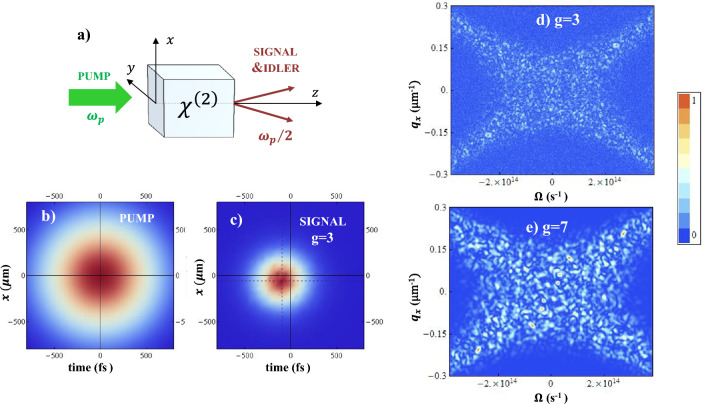
(**a**) Scheme of the PDC process. (**b**, **c**) Mean space-time distributions of the pump and of the down-converted signal, respectively, at the crystal output face. (**d**, **e**) Instantaneous signal distribution in the frequency and transverse wave-vector domain, exhibiting a broad X-shaped spectrum with small and correlated speckles, whose size increases with the gain *g*. Data taken from quantum simulations of a 2 mm BBO (Beta-Barium Borate) crystal, cut for collinear phase-matching at $\lambda _p=515$ nm, pumped by a Gaussian beam of waist $w_p=600 \, \mu$m and duration $\tau _p= 600$ fs.

Modern nonlinear optics not only enabled the first fundamental tests of quantum mechanics, but also paved the way for the advent of quantum technologies. In this context, a central role is played by parametric down-conversion (PDC), a process in which photons of a laser beam that propagates in a nonlinear $\chi ^{(2)}$ crystal occasionally split into photon pairs at lower energy (Fig 1a). The microscopic mechanism of pair creation is at the origin of a *high dimensional entanglement *^1–4^, both in the sense that paired photons are quantum correlated in different degrees of freedom (polarization, transverse position-momentum, time-frequency) and that the entanglement involves a huge number of independent modes due to the ultrabroad bandwidths of PDC. These features, which persist also at the macroscopic level of bright entangled beams, made PDC a favorite choice for quantum imaging applications (see the reviews^5–7^): the transverse entanglement of photon pairs was indeed used for pioneering realizations of ghost imaging^8^ and for quantum enhanced microscopy^9^, while the sub shot-noise spatial correlation of twin beams allowed to demonstrate high-sensitivity imaging^10, 11^, just to cite few examples. The time-frequency entanglement is at the basis of the quantum enhancement of two-photon absorption, used in entangled two-photon microscopy^12, 13^ and spectroscopy^14^.

Despite  the extensive exploitation of multimode PDC,  its theoretical description is still unsatisfactory. A  fully space-time  analytical model exists^15^ only for the low-gain regime of spontaneous PDC (SPDC), and it is widely-used to describe the quantum correlation of photon pairs^1–3^. On the other side, the bright twin beams generated by high-gain PDC are becoming, for obvious reasons, more and more attractive for quantum enhanced technologies. However, the description of their spatio-temporal correlation needs to resort to time-consuming numerical simulations^16^ or to the nonphysical limit of a plane-wave monochromatic pump^15–18^.  The few exceptions include the generalized Bogoliubov relations derived in^16^ (see also^19^ for a classical version of these equations), which however still need numerical approaches to extrapolate results. Purpose of this work is to present a substantially simpler and semi-analytic model for PDC, able to account for the finite duration and transverse cross-section of a pulsed pump in any gain regime. The model will be derived on the basis of heuristic arguments, and its predictions systematically compared with the results of numerical simulations, performed in the quantum domain, of the full model equations. We shall focus on those aspects which are not encompassed by the plane-wave and monochromatic pump (PWP) theory, namely:

- The correlation and coherence volumes of PDC light in the angle-frequency domain, which can be observed in the far-field of the source. A generally accepted view is that it is sufficient to substitute the Dirac-delta correlations of the PWP theory with finite peaks given by the angle-frequency spectrum of the pump laser, as predicted in the spontaneous regime. However, various experiments^20–22^ observed a substantial increase of the coherence and correlation lengths with increasing gain (see also the examples from numerical simulations in Fig. 1d,e).
- The space-time distribution of PDC light, in the near-field of the source. Again, the intuitive view is that it should superimpose to the pump pulse as it happens in the spontaneous regime, but, as we shall see, an important shrinking takes place at high or even moderate gain (Fig. 1c).

The formulation of this model is motivated both by practical and fundamental reasons. Today’s implementations of high-gain PDC are based on short (hundreds of femtoseconds) laser pulses, which by no means fit into the PWP description. In this respect, it is unclear whether pulse chirping, commonly employed in nonlinear optics, is suitable for quantum applications. On a more fundamental side, contrary to the SPDC regime^1–4^, a quantitative assessment of the global entanglement of multimode high-gain PDC is currently not easy, because the PWP model is unable to provide the number of entangled modes. This is of paramount importance in many areas, as e.g. in order to evaluate the quantum enhancement of two-photon absorption^13^.

## Theory background

Let us consider two wavepackets associated with the pump field (central frequency $\omega _p$) and with the downconverted signal ($\omega _s={\omega _p}/{2}$), which propagate inside a nonlinear $\chi ^{(2)}$ crystal of length $l_c$ forming small angles with a mean direction *z*. Let

1 $$\begin{aligned} \hat{A}_j(\vec{r}, t,z)&= \int \frac{d^2 \vec{q}}{2\pi } \int \frac{d\Omega }{\sqrt{2\pi }} e^{i \vec{q}\cdot \vec{r}} e^{ - i \Omega t } \hat{A}_j (\vec{q}, \Omega , z) \qquad j=s,p \end{aligned}$$

be their electromagnetic field operators, where $\vec{r}=x\vec{e}_x+y \vec{e}_y$ is the position in the transverse plane, $\vec{q}= q_x \vec{e}_x+ q_y \vec{e}_y$ is the transverse wave-vector, $\Omega$ is the frequency offset from the carriers, and dimensions are such that $\hat{A}_j^\dagger (\vec{r},t,z) \hat{A}_j (\vec{r},t,z)$ is a photon number per unit area and time. Their evolution along the slab is best described in an interaction picture in which the fast linear propagation is subtracted (see^18^ for details), by introducing

2 $$\begin{aligned} \hat{a}_j (\vec{q},\Omega ,z) = e^{- i k_{z j} (\vec{q},\Omega ) z} \hat{A}_j (\vec{w},z) , \qquad \text {where} \quad k_{jz} (\vec{q}, \Omega ) = \sqrt{k_j^2(\vec{q},\Omega ) -q^2} \, , \end{aligned}$$

$k_j(\vec{q}, \Omega )$ being the wave-number of the j-th wave (it depends on the direction of propagation through $\vec{q}$ only for the extraordinary wave). While simulations will consider the more general coupled Eq. (23), in the analytics we shall exploit the undepleted pump approximation in which the pump operator $\hat{a}_p(\vec{q},\Omega , z) \approx \hat{a}_p(\vec{q},\Omega , 0)$ is substituted by a c-number field. By adopting a shorthand notation, in which $\vec {\xi }: = (\vec{r}, t) \in R^3$ is the space-time vector and $\vec{w}: = (\vec{q}, \Omega )$ is its conjugate Fourier vector, with the convention for the scalar product: $\vec{w}\cdot \vec {\xi }:= \vec{q}\cdot \vec{r}- \Omega t$, the evolution of the PDC field along the slab is then described by:

3 $$\begin{aligned} \frac{\partial \hat{a}_s}{\partial z} (\vec{w}, z )&= \frac{g}{l_c} \int \frac{d^3 \vec{w}_0 }{(2\pi )^{\frac{3}{2}} } \, {\alpha }_p(\vec{w}_0 ) \, \hat{a}_s^\dagger (\vec{w}_0-\vec{w}, z) e^{-i \mathscr {D}(\vec{w}; \vec{w}_0-\vec{w}) z } , \end{aligned}$$

where: *g* is a dimensionless gain parameter, proportional to the nonlinear susceptibility, the crystal length and the pump peak amplitude; ${\alpha }_p(\vec{w})$ is the Fourier profile of the input pump field, normalized so that ${\alpha }_p(\vec {\xi }=0)=1$;

4 $$\begin{aligned} \mathscr {D}( \vec {w}; \vec {w}_0 - \vec {w})&:= k_{sz} (\vec {w}) + k_{sz} (\vec {w}_0 - \vec {w}) - k_{pz} (\vec {w}_0) \end{aligned}$$

is the phase mismatch of a down-conversion process in which a pump photon in mode $\vec{w}_0$ disappears and a photon pair is created in modes $\vec{w}$ and $\vec{w}_0 -\vec{w}$, with conservation of the energy and transverse momentum. The solution of Eq. (3) is a generalized Bogoliubov-type transformation, similar to that studied in^16^, linking in a nonlocal way the field operators at the crystal output $\hat{A}_s^{\mathrm out} (\vec{w})$ to the input vacuum fields. We shall not deal here with such a general solution, but rather focus on the two second order moments

5 $$\begin{aligned}&\Psi (\vec{w}_1, \vec{w}_2) = \left\langle \hat{A}_s^{\mathrm out} (\vec{w}_1) \hat{A}_s^{\mathrm out} ( \vec{w}_2) \right\rangle \end{aligned}$$

6 $$\begin{aligned}&G^{(1)}(\vec{w}_1, \vec{w}_2) =\left\langle \hat{A}_s^{\dagger \, \mathrm out}(\vec{w}_1) \hat{A}_s^{\mathrm out} \vec{w}_2) \right\rangle \end{aligned}$$

that for such a Gaussian process determine all the spatio-temporal statistical properties at the medium output. The first function, is the *biphoton correlation*, proportional to the probability amplitude of generating a pair of twin photons in modes $\vec{w}_1$ and $\vec{w}_2$. The second one is the ubiquitous coherence function, describing the autocorrelation of light when twin photons are not detected together, for example, by measuring only one side of the spectrum with respect to the central frequency. All higher order moments can be expressed in terms of the second order ones. For  example the correlation of the light intensity $\hat{I} (\vec{w}) = \hat{A}_s^{\dagger \, \mathrm out}(\vec{w}) \hat{A}_s^{\mathrm out} (\vec{w})$ reads: $\langle : \hat{I} (\vec{w}_1) \hat{I} (\vec{w}_2) :\rangle - \langle \hat{I} (\vec{w}_1) \rangle \langle \hat{I} (\vec{w}_2) \rangle = | G^{(1)}(\vec{w}_1, \vec{w}_2) |^2 + \left| \Psi (\vec{w}_1, \vec{w}_2) \right| ^2$.

Goal of the next sections will be to determine the functional form of $\Psi$ and $G^{(1)}$, holding under specific approximations. Before that, let us remind the well-known results of the plane-wave pump model^15^, in which ${\alpha }_p(\vec{w}) \rightarrow (2 \pi )^{3/2} \delta (\vec{w})$, and

7 $$\begin{aligned}&\Psi (\vec{w}_1, \vec{w}_2) {\mathop { \rightarrow }\limits ^{ PWP}} \delta (\vec{w}_1 + w_2) \, U(\vec{w}_1) V(-\vec{w}_1) e^{ik_p l_c} \qquad&G^{(1)}(\vec{w}_1, \vec{w}_2) {\mathop { \rightarrow }\limits ^{ PWP}} \delta (\vec{w}_2 - \vec{w}_1) \, \left| V (\vec{w}_1)\right| ^2 \, . \end{aligned}$$

where $U(\vec{w}) = \cosh { \left[ \Gamma (\vec{w}) \right] } + i\frac{D_{{\textsc {pw}}}(\vec{w})l_c}{ 2 \Gamma (\vec{w})} \sinh \left[ \Gamma (\vec{w}) \right]$ ; $V(\vec{w}) = g \frac{\sinh \left[ \Gamma (\vec{w}) \right] }{ \Gamma (\vec{w})}$, with $\Gamma (\vec{w})= \sqrt{g^2 - \frac{[D_{{\textsc {pw}}}(\vec{w})l_c]^2}{4} }$. The two functions are strongly peaked in the regions where the plane-wave phase mismatch

8 $$\begin{aligned} D_{{\textsc {pw}}}(\vec{w}) := \mathscr {D}(\vec{w}, -\vec{w}) \end{aligned}$$

vanishes. In the example of Fig. 1, where parameters are chosen for collinear phase matching, it corresponds to the broad X-shape of the Fourier spectrum. The PWP theory has the undoubted merit of describing very well the angle-frequency distribution of PDC light in any gain condition^23–26^, and the existence of non-classical correlations between conjugated Fourier modes (see e.g.^5, 17, 27^). An obvious drawback are the Dirac-delta correlations in the Fourier domain, direct consequence of assuming a homogeneous distribution in the space-time domain.

## Results

### Our proposal: the quasi-stationary model

With the aim of formulating a model valid both in the low and high gain of PDC, but without the heavy limitations of the plane-wave pump approximation, we focus on a sufficiently narrowband pump, such that the pump Fourier profile dies out on a faster scale than the phase matching function:

9 $$\begin{aligned} \mathscr {D}(\vec{w},\vec{w}_0-\vec{w}) l_c \simeq D_{\textsc {pw}}(\vec{w}) l_c + \Omega _0 \tau _{\textsc {gvm}}+ q_{0x} {\l}_{\textsc {woff}}\rightarrow D_{\textsc {pw}}(w) l_c \qquad \forall \, \vec{w}_0 \text { inside the pump spectrum}. \end{aligned}$$

where the Taylor expansion (19) has been used. Here $\tau _{\textsc {gvm}}=\frac{ l_c}{v_{gs}} -\frac{ l_c}{v_{gp}}$ and ${\l}_{\textsc {woff}}=- l_c \rho _p$ are the overall temporal and spatial walk-off occurring between the signal and the pump during propagation (Section “Methods”), due to their group velocity mismatch and the walk-off of the Poynting vector, respectively. Strictly speaking the limit (9) requires a pulse of duration $\tau _p \gg \tau _{\textsc {gvm}}$ and waist $w_p \gg {\l}_{\textsc {woff}}$. In the spontaneous regime of PDC, these conditions ensure that both the biphoton correlation and the coherence function factorize into the product of two functions with different scales of variation^24, 25^. In high-gain we do not have such explicit expressions. Our approach will be to assume that in any regime the $\Psi$ and $G^{(1)}$ factorize in the product of a fast decaying correlation peak and a slowly varying envelope. This ansatz, to which we shall refer as *Quasi-Stationary* (QS) approximation, corresponds to a configuration where small speckles are observed inside a broader spectral distribution, as depicted by the simulation of Fig. 1 and observed in several high-gain experiments^21, 23, 26, 28^ We expect that such separation of decay scales holds in the limit (9), but we shall verify this point a posteriori. As for the specific choices of envelopes and correlations, we set:

10a $$\begin{aligned}&\Psi (\vec{w}_1, \vec{w}_2) = \mu _{corr} (\vec{w}_1+\vec{w}_2) \, U(\vec{w}_1) V(\vec{w}_1) e^{i k_p l_c} \, , \; \text{ with }\\&\mu _{corr} (\vec{w}) = \int \frac{d^3 \xi }{(2\pi )^3} e^{-i \vec{w}\cdot \vec {\xi }} \, \frac{ \sinh {[ 2 g |{\alpha }_p(\vec {\xi }-\vec {\xi }_M)| ]} }{ \sinh {2g}} e^{i \phi _p (\vec {\xi }-\vec {\xi }_M)} \end{aligned}$$

where $\phi _p (\vec {\xi })= \arg {[ {\alpha }_p(\vec {\xi })]}$, and *U* and *V* are the functions of the PWP model (7), and

10b $$\begin{aligned}&G^{(1)}(\vec{w}_1, \vec{w}_2) = \mu _{coh} (\vec{w}_2-\vec{w}_1) \, \left| V(\vec{w}_1) \right| ^2 \; \text{ with } \\&\mu _{coh} (\vec{w}) = \int \frac{d^3 \xi }{(2\pi )^3} e^{-i \vec{w}\cdot \vec {\xi }} \, \frac{ \sinh ^2 {[g |{\alpha }_p(\vec {\xi }- \vec {\xi }_M)| ]} }{ \sinh ^2{g}} \end{aligned}$$

In these formulas $\vec{r}=0$ and $t=0$ are the coordinates of the pump center at the crystal output. Accordingly,

11 $$\begin{aligned} \vec {\xi }_M= \frac{{\l}_{\textsc {woff}}}{2} \vec{e}_x+ \frac{\tau _{\textsc {gvm}}}{2} \vec{e}_t \end{aligned}$$

represents, as we shall see, an offset (in space and time) between the signal and pump beams at the crystal output face. Clearly, in the limit of a homogeneous and stationary pump in which ${\alpha }_p(\vec {\xi })=1$, the PWP results of Eq. (7) are recovered. On the other hand, the functions $\mu _{corr}$ and $\mu _{coh}$ that replace the Dirac-delta of the PWP model have been chosen on the basis of an other notable limit, namely that of a *thin crystal*, in which one takes $D_{\textsc {pw}}(\vec{w}) l_c \approx 0$ for all the modes of interest. Then, the propagation Eq. (3) can be easily solved (Section “Methods”), and the  equations (10a) and (10b) converge asymptotically to the results thereby obtained. Notice that the thin crystal limit, which discards the contribution of modes which are not phase-matched, is complementary to the PWP limit, because it assumes a homogeneous distribution in the Fourier domain.

### Correlation and coherence volumes in the Fourier space

The QS model of Eqs.(10) merges the ”good” features of the plane-wave pump and thin crystal approximations, by giving asymptotically the same results, but being free from their ill behaviors.

Said that, then a good question is whether this model makes correct predictions in realistic conditions, and what are their limits of validity. To this end, we shall compare the predictions of the QS model with the results of numerical stochastic simulations of the complete model (23).

Let us focus on a real and symmetric pump, i.e. a pump that is not chirped and that satisfies ${\alpha }_p(-\vec {\xi }) ={\alpha }_p(\vec {\xi })$. Then, the widths of the spectral correlations (10a) and (10b) can be straightforwardly evaluated. By defining

12 $$\begin{aligned} &F_{ corr} (\vec {\xi }) =\frac{ \sinh {[ 2 g |{\alpha }_p(\vec {\xi })| ]} }{ \sinh {2g}}, \qquad \qquad&F_{ coh} (\vec {\xi }) = \frac{ \sinh ^2 {[ g |{\alpha }_p(\vec {\xi })| ]} }{ \sinh ^2{g}} \end{aligned}$$

we have

13 $$\begin{aligned}&\left| \mu _\beta (\vec{w}) \right| = \int \frac{ d^3 \xi }{(2 \pi )^3} e^{-i \vec {\xi }\cdot \vec{w}} \, F_\beta (\vec {\xi }) ; \qquad&\int d^3 w \, \left| \mu _\beta (\vec{w}) \right| = \int d^3 \xi F_\beta (\vec {\xi }) \, \delta (\vec {\xi }) = F_\beta (0) =1 \qquad (\beta =corr,\, coh ) \end{aligned}$$

Hence, each $\left| \mu _\beta \right|$ defines a normalized distribution, having $F_{\beta }$ as its characteristic function.  The mean values vanish for symmetry reasons, which gives the usual result that the peak of the coherence is at $\vec{w}_2= \vec{w}_1$, while the biphoton correlation is peaked at $\vec{w}_2= -\vec{w}_1$.  The widths of the distributions can be evaluated as

14 $$\begin{aligned} \begin{aligned} \langle w_i^2 \rangle _\beta -\langle w_i \rangle _\beta ^2&= \int d^3 w \left| \mu _{\beta } (\vec{w}) \right| \, w_i^2 = \int d^3 w \int \frac{d^3 \xi }{(2\pi )^3} \left[ -\frac{\partial ^2}{\partial \xi _i^2} e^{-i \vec {\xi }\cdot \vec{w}}\right] F_\beta (\vec {\xi }) = - \left. \frac{ \partial ^2 F_\beta (\vec {\xi })}{\partial \xi _i^2} \right| _{\vec {\xi }=0} \end{aligned} \end{aligned}$$

where $w_i$ and $\xi _i \,$ ($i=1,2,3$) denote the components of the vectors $\vec{w}$ and $\vec {\xi }$, and integration by parts has been performed twice. By focusing on a a Gaussian pump of the form ${\alpha }_p(\vec {\xi }) = \exp { ( -\sum _i \frac{\xi _i^2}{ {\Delta _p}_i^2} )}$ where ${\Delta _p}_i= w_{px}, w_{py}, \tau _p$ are the 1/e widths in the spatial and temporal directions, we have $\langle w_i^2 \rangle _{corr} = \frac{2g}{\tanh (2g)} \frac{2}{{\Delta _p}_i^2}$, and $\langle w_i^2 \rangle _{coh} = \frac{2g}{\tanh (g)} \frac{2}{{\Delta _p}_i^2}$. The QS model provides in this way simple and explicit formulas for the correlation and coherence lengths in the Fourier space, defined here as the standard deviations of the respective distributions $|\mu _{\beta }|$

15a $$\begin{aligned}{}&\Delta w_{i \, corr} = \sqrt{\langle w_i^2 \rangle _{corr} } = \sqrt{ \frac{4g}{\tanh (2 g)} } \frac{1}{{\Delta_{pi}}} \end{aligned}$$

15b $$\begin{aligned}{}&\Delta {w_i}_{ coh} = \sqrt{\langle w_i^2 \rangle _{coh} } = \sqrt{ \frac{4g}{\tanh (g)} } \frac{1}{{\Delta _{pi}}} \end{aligned}$$

**Figure 2:**
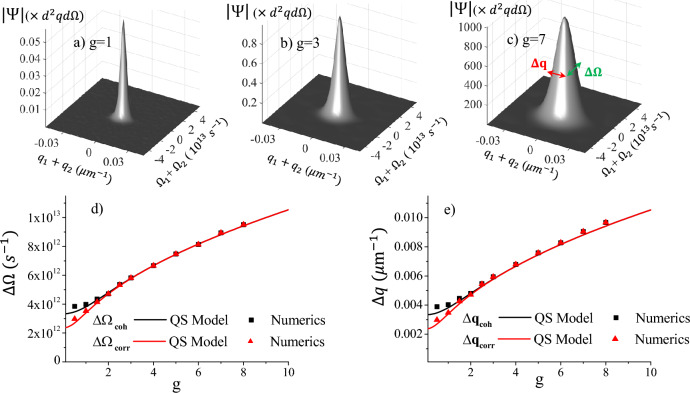
”Long” pump $\tau _p= 600$ fs, $w_p=600 \, \mu$m. (**a**–**c**): Examples of the biphoton correlation ($\left| \Psi \right|$, multiplied by the volume $d^2q d\Omega$ of the simulation pixel), numerically calculated by averaging over 3000–10000 stochastic realizations of Eq. (23), showing the increase of the correlation volume with gain. (**d**) Spectral width (standard deviation) $\Delta \Omega$ of $\left| \Psi \right|$ (red) and of $| G^{(1)}|$ (black) as a function of the gain *g*. Solid lines: predictions of the QS model, according to Eq. (15). Symbols: results of simulations. (**e**) Same as (**d**), but for the width along *q*.

The two curves are plotted by the solid lines in Fig. 2. For large gains, the correlation and the coherence functions have the same width, which increases with the gain as $\Delta w_{i\beta } \sim \sqrt{4g}/{\Delta _p}_i$. This is in nice agreement with the experimental findings^21, 22^, where a $g^{1/2}$ growth of the size of the speckles was observed in the angle-frequency domain of high-gain PDC. At small gains, the two curves separate, with $\Delta w_{i\, corr} \rightarrow \sqrt{2} /{\Delta _p}_i$, and $\Delta w_{i\, coh} \rightarrow 2/{\Delta _p}_i$, which are just the inverse of the standard deviations of the pump amplitude and intensity, respectively. Actually, we notice that in the limit $g\ll 1 \,$ $\mu _{corr} (\vec{w})$ becomes the Fourier transform of the pump *amplitude*, while $\mu _{coh} (\vec{w})$ becomes the Fourier transform of the pump *intensity*, in agreement with the results known for spontaneous PDC^24, 25^.

**Figure 3:**
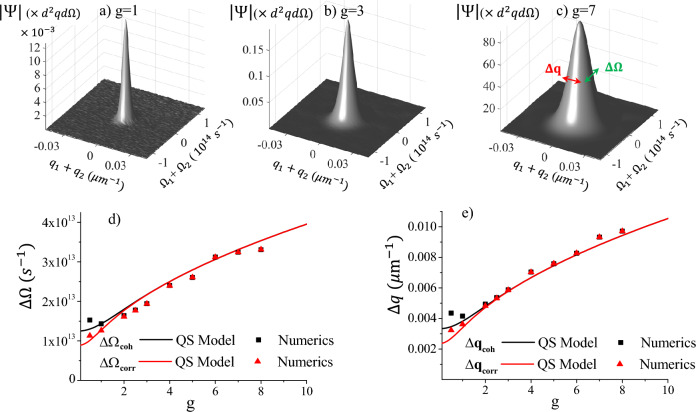
”Short” pump $\tau _p= 160$ fs, $w_p=600 \, \mu$m. Same as Fig. 3, except that up to $10^5$ stochastic realizations were necessary to obtain the numerical results, due to the high noise. Notice the fair agreement with the QS model predictions, despite $\tau _p < |\tau _{\textsc {gvm}}|$.

Superimposed to the analytical predictions, the symbols show for comparison the results of the complete model, obtained by stochastic 2D+1 simulations of the propagation Eq. (23), performed in the Wigner representation (Section “Methods”). We considered a 2mm BBO crystal cut for degenerate and collinear phase matching at $\lambda _p=515\,$ nm; in these conditions, the group velocity delay is $\tau _{\textsc {gvm}}=- 185.6\,$ fs, while the lateral walk-off is ${\l}_{\textsc {woff}}=-113 \, \mu$m. The pump duration and waist ($\tau _p=600$ fs and $w_p = 600\, \mu$m, respectively) were chosen reasonably larger than these values, in order to meet the condition (9) of a nearly plane-wave pump. Indeed, for these parameters the predictions of our simple QS model appear in excellent agreement with the results of simulations. Conversely, Fig. 3 shows the results of simulation of PDC from a much shorter pulse $\tau _p=160\,$fs, which is well outside the expected conditions of validity of the QS model. Nevertheless, the agreement with the QS model is still fairly satisfactory: numerical results are more scattered than in Fig. 2, but follow nicely the behaviors of a $\sqrt{4g}$ growth at high gain, and a bifurcation at low gain. At low gain the agreement is not perfect, but this may also be due to the impact of residual noise.

### Exponential narrowing of the space-time distributions

The growth of the correlation and coherence volumes in the Fourier domain with increasing gain is strictly linked to a progressive narrowing of the space-time distribution of PDC photons at the medium output: at low gain these simply follow the profile of the pump, because at each point of the medium the photon pairs are generated independently with probability proportional to $|\alpha _p (\vec{r},t)|^2$. At high gain the signal distribution becomes much narrower, because of stimulated processes that exponentially grow in the central region of the pump peak. More formally, by Fourier transforming the spectral correlations in Eq. (10), one obtains

16a $$\begin{aligned}{}&\Psi (\vec {\xi }_1, \vec {\xi }_2) = \left\langle \hat{A}_s^{\mathrm out} (\vec {\xi }_1) \hat{A}_s^{\mathrm out} (\vec {\xi }_2) \right\rangle = F_{corr} (\vec {\xi }_1-\vec {\xi }_M) \int \frac{d^3 w}{ (2 \pi )^3} \, e^{i (\vec {\xi }_2 -\vec {\xi }_1) \cdot \vec{w}} U(\vec{w}) V(\vec{w}) \end{aligned}$$

16b $$\begin{aligned}{}&G^{(1)}(\vec {\xi }_1, \vec {\xi }_2) = \left\langle \hat{A}_s^{\dagger \, \mathrm out}(\vec {\xi }_1) \hat{A}_s^{\mathrm out} (\vec {\xi }_2) \right\rangle = F_{coh} (\vec {\xi }_1-\vec {\xi }_M) \int \frac{d^3 w}{ (2 \pi )^3} \, e^{i (\vec {\xi }_2 -\vec {\xi }_1) \cdot \vec{w}} | V(\vec{w})|^2 \end{aligned}$$

The Fourier integrals at r.h.s.of Eqs. (16a) and (16b) define two spatio-temporal correlation peaks centered around $\vec {\xi }_2=\vec {\xi }_1$, known in the literature as X-entanglement^24–26^ and X-coherence^23^, respectively,  because of their particular shape. We shall not deal here with these aspects, already extensively studied, but focus on the envelopes $F_\beta$. In the QS model, these have well defined expressions also at high-gain, that cannot be described within the PWP model used e.g. in^25^. In particular, the QS model provides the mean spatio-temporal distribution of the PDC photons in any gain regime:

17 $$\begin{aligned} \left\langle \hat{A}_s^{\dagger \, \mathrm out}(\vec {\xi }) \hat{A}_s^{\mathrm out} (\vec {\xi }) \right\rangle = G^{(1)}(\vec {\xi },\vec {\xi })= \frac{\sinh ^2 [ g |{\alpha }_p(\vec {\xi }-\vec {\xi }_M)|] }{\sinh ^2 (g)} \int \frac{d^3 w}{ (2 \pi )^3} | V(\vec{w})|^2 \end{aligned}$$

**Figure 4:**
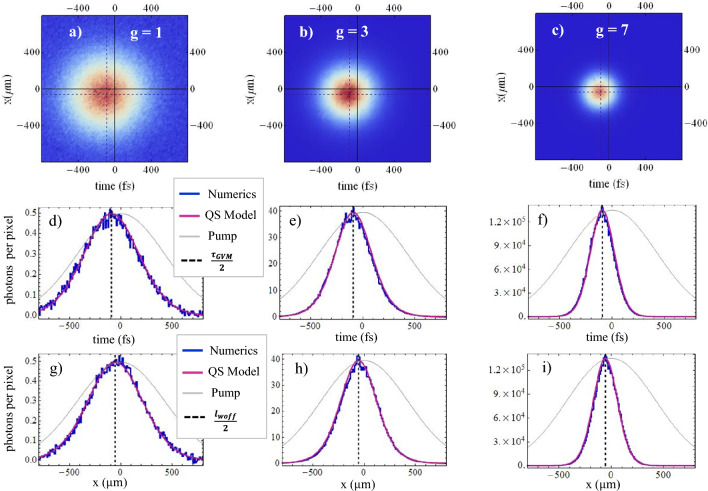
Space-time distributions (mean photon number on the pixel of the numerical grid) of the PDC light at the crystal output, evidencing the narrowing of the signal beam with increasing gain. ”Long” pump $\tau _p= 600$ fs, $w_p=600 \, \mu$m. First row: 2-D plots from simulations. Second and third row: sections along time and space, respectively. Superimposed to the numerics (blue), the red curves show the predictions (17) of the QS model, multiplied by a fit parameter, while the gray lines show the pump profile. $g=1$ in (**a**), (**d**) and (**g**). $g=3$ in (**b**), (**e**) and (**h**). $g=7$ in (**c**), (**f**) and (**i**).

**Figure 5:**
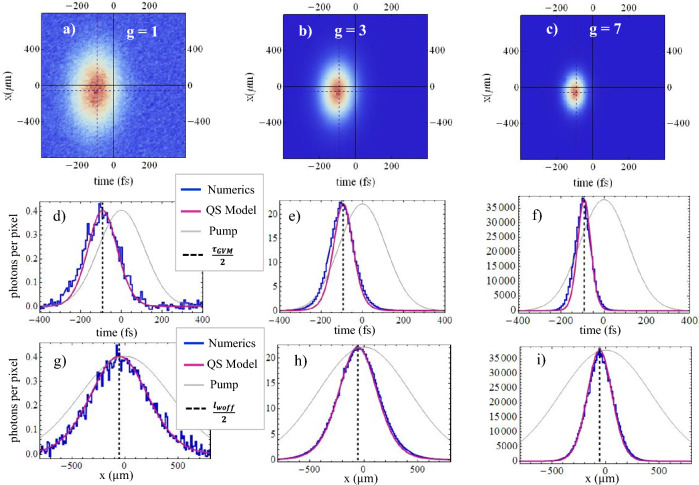
Same as Fig. 4, but for a ”short” pump pulse $\tau _p= 160$ fs, $w_p=600 \, \mu$m.

The behavior of this function is shown by the red lines in Figs. 4 and 5. The QS model predicts that the signal pulse is significantly narrower and shorter than the pump pulse (gray lines) even at moderate gain as $g=1$; in addition, it predicts that it appears at the medium output delayed (actually anticipated) by a an amount $\frac{\tau _{\textsc {gvm}}}{2}$ and laterally shifted by $\frac{{\l}_{\textsc {woff}}}{2}$. The blue lines are instead the results of quantum simulations of the complete model, which fully incorporate the effects of temporal and spatial walk-off as well as of dispersion and diffraction. As already observed for Fig. 2, the QS model, despite its highly simplified formulation, provides an excellent description of the process.

The fit between analytics and numerics is almost perfect for the long pump $\tau _p=600$ fs in Fig. 4, but remains very satisfactory also for the short pump $\tau _p=160$ fs in Fig. 5.

**Figure 6:**
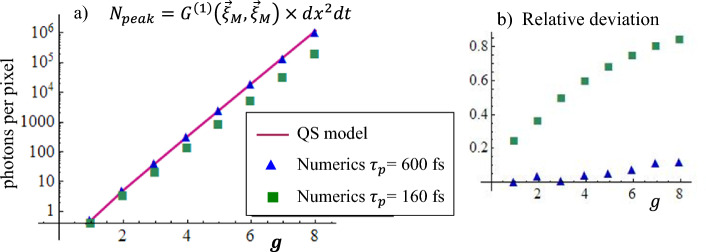
(**a**) Mean number of photons at the peak of the signal pulse $\langle \hat{A}_s^{\dagger \, \mathrm out}(\vec {\xi }_M) \hat{A}_s^{\mathrm out} (\vec {\xi }_M) \rangle \times dx^2 dt$ (units are photons per numerical pixel). The red line is the QS model prediction in Eq. (17), where the integral was calculated via a discrete sum over the same numerical grid of the simulations. The symbols are the results of simulations. (**b**) Relative deviation between analytic predictions and numerical results

In this regard, however, it should be noted that the graphs were produced with the aid of a fit parameter that multiplies the analytic function (17). This parameter was close to unity for the long pulse, but significantly smaller for the short one. In fact, as illustrated by Fig. 6, the QS model is able to provide a reasonable approximation of the mean number of generated photons, within a $10 \%$ error, only in the case of the long pump pulse. On the contrary, it essentially fails in the case of a pulse shorter or on the same order as $\tau_{\textsc {gvm}}$. This is predictable, as the QS model takes into account the effects of the spatial and temporal walk-off between the pump and signal only through a rigid traslation of the two distributions: as is known, however, the loss of overlap between the two beams during propagation, due to their different group velocities and the spatial walk-off of the extraordinary pump, reduces the efficiency of the stimulated conversion processes. Nevertheless, we note that even in the case of $\tau _p = 160$ fs where the group velocity delay $\tau _{\textsc {gvm}}=-185$ fs causes an important loss of overlap between the signal and pump (see Fig. 5d,e and f), the QS model still provides a very efficient description of the size and shape of the signal beam.

## Discussion

We formulated a semi-analytical model describing pulsed PDC in any gain regime and verified its predictions through simulations of the full model equations. The rationale behind the formulation of this model is: i) That it converges asymptotically to the appropriate limits in the cases where the solution of the evolution Eq. (3) is known, i.e. the limits of PWP, thin crystal and $g \rightarrow 0$. ii) That it is substantially simpler than analogous models previously formulated^16^, still retaining the essential features of the process. iii) That different results can be accessed via analytical calculations. Indeed, it is a strength of our QS model to provide simple analytic formulas, such as Eq. (15) for the correlation and coherence volumes, which hold in both the spontaneous and stimulated PDC regimes, as it was verified by comparison with the full model (Figs. 2 and 3).

The model essentially neglects the effects of the spatial and temporal walk-off between the pump and signal, except for a rigid translation of the two distributions: as such we expect it to be valid only for pump pulses substantially longer than $\tau _{\textsc {gvm}}$ and broader than ${\l}_{\textsc {woff}}$. Surprisingly, we verified that its predictions regarding the shape and size of the Fourier coherence and correlation, as well as the space-time distribution of PDC, remain valid even well outside these boundaries, as e.g. for a 160 fs pulse propagating in 2 mm crystal. Clearly, aspects such as the loss of efficiency of the stimulated PDC due to the walk-off are overlooked; these would require more sophisticated descriptions^29^, lacking however the immediacy and simplicity of the model here presented.

## Methods

### Expansion of phase matching

We expand the phase matching defined by Eq. (4) in Taylor series of the pump variable $\vec{w}_0 = (q_{0x},q_{0y}, \Omega _0)$. Provided that the duration and cross section of the pump pulse are large enough to make its diffraction and dispersion along the medium negligible (and/or the crystal is short enough), quadratic and higher order terms can be neglected, so that:

18 $$\begin{aligned} \mathscr {D}( \vec{w}; \vec{w}_0 - \vec{w}) - \mathscr {D}( \vec{w}; - \vec{w}) \simeq \left[ \mathbf {\vec\nabla } k_{sz} (-\vec{w})-\mathbf {\vec \nabla } k_{pz} (0) \right] \cdot \vec{w}_0 \simeq \left( k^\prime _s -k^\prime _p - k''_s \Omega \right) \Omega _0 - \left( \rho _p \vec{e}_x+\frac{\vec{q}}{k_s} \right) \cdot \vec{q}_0 \end{aligned}$$

where in the second passage only the leading terms of the expansion of $\mathbf {\nabla } k_{sz} (-\vec{w})$ around $\vec{w}=0$ (the central frequency and the collinear direction) have been retained.  Here $k^\prime _j= \frac{d k_j}{d\Omega } ({\vec{w}=0} )=\frac{1}{v_{gj}}$, where $v_{gs}$ and $v_{gp}$ denote the group velocities of the signal and pump wave-packets; $k''_s = \frac{d^2 k_s}{d\Omega ^2} (\vec{w}=0)$ ; $\rho _p \approx \frac{d k_p}{dq_x}$ is the *walk-off* angle of the Poynting vector of the extraordinary pump, assumed here in the x-direction. The term $k''_s \Omega \Omega _0$ is usually negligible, unless special points are considered: for example, with our parameters it would be significant only for $|\Omega |$ as large as $|k'_s-k'_p|/k''_s = 2. 10^{15} s^{-1}$, which is larger than $\omega _s$. For a very focused pump, the term $\frac{\vec{q}\cdot \vec{q}_0}{k_s}$ may originate the so-called *hot spots*^30^, but in any case it is relevant only close to a specific angle $q_x/k = -\rho _p$, which we do not consider in our analysis (for our BBO $\rho _p=-3.2^\circ$). Therefore, under basically the requirement that diffraction and dispersion of the pump along the medium can be neglected, we do not make a big error by writing

19 $$\begin{aligned} \mathscr {D}( \vec{w}; \vec{w}_0 - \vec{w}) l_c = \left[ D_{\textsc {pw}}(\vec{w}) + \left( k^\prime _s -k^\prime _p \right) \Omega _0 - \rho _p \vec{e}_x\cdot \vec{q}_0 \right] l_c = D_{\textsc {pw}}(\vec{w})l_c + \tau _{\textsc {gvm}}\Omega _0 + {\l}_{\textsc {woff}}q_{0x} \end{aligned}$$

### Thin crystal solution

We consider here a particular limit, in which *g* is finite, but the crystal is thin enough that all the terms of Eq. (19) are negligible, so that one can set

20 $$\begin{aligned} \mathscr {D}(\vec{w}, \vec{w}_0-\vec{w}) l_c \approx 0 \end{aligned}$$

for all the modes of interest. By back transforming the propagation Eq. (3) into direct space, one obtains the simple parametric equation

21 $$\begin{aligned} \frac{\partial \hat{a}_s}{\partial z} (\vec {\xi }, z )&= \frac{g}{l_c} \, {\alpha }_p(\vec {\xi }) \, \hat{a}_s^\dagger (\vec {\xi }, z) , \end{aligned}$$

in which space-time points are not coupled, and the parametric gain is modulated by the pump spatio-temporal profile. The solution can be written as : $\hat{a}_s^{out} (\vec {\xi }) = \cosh { \left[ g |\alpha _p (\vec {\xi }) \right] } \hat{a}_s^{in} (\vec {\xi }) + e^{i \phi _p (\vec {\xi })} \sinh { \left[ g |\alpha _p (\vec {\xi }) \right] } { \hat{a}_s^{in\, \dagger }} (\vec {\xi })$, which immediately gives: $\langle \hat{a}_s^{out} (\vec {\xi }) \hat{a}_s^{out} (\vec {\xi }') \rangle = \delta (\vec {\xi }-\vec {\xi }') \frac{1}{2} \sinh { \left[ 2 g |\alpha _p (\vec {\xi })| \right] } e^{i \phi _p (\vec {\xi })}$, and $\langle \hat{a}_s^{out\, \dagger } (\vec {\xi }) \hat{a}_s^{out} (\vec {\xi }') \rangle = \delta (\vec {\xi }-\vec {\xi }') {\sinh ^2{ \left[ g |\alpha _p (\vec {\xi })| \right] }}$. We stress that in principle these are not the second order moments in the direct space, because the lowercase operators $\hat{a}_s$ are connected to the actual photonic operator $\hat{A}_s$ by the Fourier space transformation (2). However, in the spirit of the thin crystal approximation (20), the propagation phase factors can be neglected, setting $l_c [k_{sz} (\vec{w}) + k_{sz}(\vec{w}') ] \approx l_c k_p$ and $l_c [k_{sz} (\vec{w}) - k_{sz} (\vec{w}') ] \approx 0$. Therefore, the second order moments in the Fourier space can be obtained by Fourier transforming the above results, as

22 $$\begin{aligned} & \Psi (\vec{w}, \vec{w}') \approx \, e^{ik_p l_c} \int \frac{d^3 \xi }{(2\pi )^3} e^{-i ( \vec{w}+\vec{w}') \cdot \vec {\xi }} \, \frac{ 1}{2} \sinh {[ 2 g |{\alpha }_p(\vec {\xi })| ]} e^{i \phi _p (\vec {\xi })} \\ & G^{(1)}(\vec{w}, \vec{w}') \approx \int \frac{d^3 \xi }{(2\pi )^3} e^{-i ( \vec{w}' -\vec{w}) \cdot \vec {\xi }} \, \sinh ^2 {[ g |{\alpha }_p(\vec {\xi })| ]} \end{aligned}$$

These formula coincide with those of the QS model, when the thin crystal limit of Eq. (10) is taken, because in the limit (20), $U (\vec{w}) \rightarrow \cosh {g}$ , $V(\vec{w}) \rightarrow \sinh {g}$, and also the displacement $\vec {\xi }_M$ becomes negligible on the pump scale.

At this point one might ask where the precise value $\vec {\xi }_M = \frac{{\l}_{\textsc {woff}}}{2} \vec{e}_x+ \frac{\tau _{\textsc {gvm}}}{2 } \vec{e}_t$ comes from, also considering that it coincides with what observed in the simulations even for very short pump pulses. Actually, this result was obtained in the context of a slightly more sophisticated model for a thin crystal^29^, which for reasons of brevity is not presented here. The analysis in^29^ fully retains the effects of the term $\Omega _0 \tau _{\textsc {gvm}}+ {\l}_{\textsc {woff}}q_{0x}$ in the phase matching expansion (19), and indeed demonstrates that the signal appears at the crystal output displaced by $\vec {\xi }_m$ with respect to the pump. For reasonably small values of $\Delta = [ (\frac{\tau _{\textsc {gvm}}}{\tau _p})^2 + (\frac{{\l}_{\textsc {woff}}}{w_p})^2 ]^{1/2}$ , this rigid displacement is basically the only deviation from the more naive analysis of this section, and for this reason it was incorporated into the QS model. For larger values of $\Delta$, there is also^29^ the expected loss of efficiency due to the lack of overlap between the two co-propagating beams, which instead is neglected by the QS model.

### Numerical simulations

We considered the nonlinear propagation equations for the coupled signal and pump operators:

23a $$\begin{aligned} \frac{\partial \hat{a}_s}{\partial z} (\vec{w}_s, z )&= \chi \int \frac{d^3 \vec{w}_p }{(2\pi )^{\frac{3}{2}} } \hat{a}_p(\vec{w}_p ,z) \hat{a}_s^\dagger (\vec{w}_p -\vec{w}_s, z) e^{-i \mathscr {D}(\vec{w}_s, \vec{w}_p-\vec{w}_s) z } \end{aligned}$$

23b $$\begin{aligned} \frac{\partial \hat{a}_p }{\partial z} (\vec{w}_p, z )&= -\frac{ \chi }{2} \int \frac{d^3 \vec{w}_s }{(2\pi )^{\frac{3}{2}} } \hat{a}_s(\vec{w}_s ,z) \hat{a}_s(\vec{w}_p -\vec{w}_s, z) e^{i \mathscr {D}(\vec{w}_s, \vec{w}_p -\vec{w}_s) z } \end{aligned}$$

Simulations of these equations were performed in the framework of the quantum to classical correspondence, in the Wigner representation, which provides the symmetrically ordered moments of observables. More precisely, we used a truncated Wigner representation^31, 32^, in which the quantum operators are replaced by c-number fields that evolve with equations formally identical to Eq. (23), and the quantum noise contributes only through the vacuum input fluctuations. In our simulations, they are modeled by taking Gaussian white noise as initial condition for the signal. The input pump, which is assumed to be a high-intensity coherent pulse, is modeled by a Gaussian profile in space and time. For all the considered simulation parameters, we found that the pump was nearly undepleted.

We considered PDC in a 2mm BBO crystal, tuned for type I e-oo collinear phase-matching at degeneracy when pumped by a laser at $\lambda _p=515$nm (angle of propagation $\sim 23.29^\circ$ with the optical axis). In these conditions the signal spectrum exhibits the characteristic X-shape shown in Fig. 1d,e. Numerical integration is performed through a second-order pseudo-spectral (split-step) method^33^. Temporal dispersion and diffraction are taken into account at any order by using the complete Sellmeier relations for the refractive index of the material^34^. Since our results need averages performed over a large number of independent realizations (up to $10^5$ for small *g*) , which are extremely time-consuming, we performed 2D+1 simulations, restricting to one transverse spatial dimension, i.e. the one along the walk-off direction. Our numerical grid is 512$\times$512 pixels in the $q_x$ and $\Omega$ directions, spanning the symmetric bandwidths $-0.3\,{\upmu \text{m}}^{-1}<q_x<0.3\,{\upmu \text{m}}^{-1}$, and $-3.84\times 10^{14}\textrm{s}^{-1}<\Omega <3.84\times 10^{14}\textrm{s}^{-1}$ ($850\textrm{nm}<\lambda <1304$nm) around the collinear direction and the degenerate frequency (see Fig. 1). In the $q_y$ direction we took a single pixel, which may simulate a narrow slit in the far-field of the source that selects a single mode around $q_y=0$, as e.g. done for frequency-resolved detection by means of an imaging spectrometer^21^.

The biphoton correlation and the coherence function defined by Eqs. (5) and (6) were evaluated by performing ensemble averages over a large number (from $10^3$ to $10^5$, depending on the gain) of independent realizations, obtained by integrating the propagation Eq. (23) starting from independently and randomly generated initial conditions. In order to boost the convergence rate of the simulations we also exploited the translation invariance of the field statistics in the central region of the Fourier plane where the spectrum is nearly uniform. We considered the rectangular region $R=[-0.1\,{\upmu \text{m}}^{-1},0.1\,{\upmu \text{m}}^{-1}]\times [0,1.2\, 10^{14} \textrm{s}^{-1}]$, containing $M=13338$ pixels and in each realization calculated the discrete convolutions $C_{corr}(\vec{w}_k)= \frac{1}{M}\sum _{\vec{w}_j} A_s^{out}(\vec{w}_j )A_s^{out}(-\vec{w}_j +\vec{w}_k)$ and $C_{coh}(\vec{w}_k)= \frac{1}{M}\sum _{\vec{w}_j} A_s^{\star \, out}(w_j )A_s^{out}(\vec{w}_j +\vec{w}_k)$, where the $\vec{w}_j$ run over the pixel coordinates inside R. Examples of the biphoton correlation obtained in this way are shown in Figs.2 and 3. The standard deviations $\Delta \Omega$ and $\Delta q$ here reported were evaluated by using the $\left| \Psi \right|$ and $| G^{(1)}|$ thereby obtained (after correcting the $G^{(1)}$ in order to pass from symmetric to normal ordering). A delicate point is represented by the residual noise of the Wigner simulation, still visible e.g. in Fig. 3a, that may artificially enhance $\Delta \Omega$ and $\Delta q$: for this reason the variances along each Fourier coordinate were calculated over a reduced region, covering 8 times the standard deviations (15) of the QS model. This number seemed a good compromise between the need to cover the entire peak and to avoid including too much residual noise.
